# Lymphocyte‐C‐Reactive Protein Ratio: Impact on Prognosis of Patients Following Resection of Primary Liver Cancer

**DOI:** 10.1002/wjs.12675

**Published:** 2025-06-17

**Authors:** Abdullah Altaf, Andrea Baldo, Mujtaba Khalil, Zayed Rashid, Miho Akabane, Shahzaib Zindani, Azza Sarfraz, Andrea Ruzzenente, Luca Aldrighetti, Todd W. Bauer, Hugo P. Marques, Guillaume Martel, Irinel Popescu, Mathew J. Weiss, Minoru Kitago, George Poultsides, Shishir K. Maithel, Vincent Lam, Tom Hugh, Ana Gleisner, Feng Shen, François Cauchy, Bas G. Koerkamp, Itaru Endo, Timothy M. Pawlik

**Affiliations:** ^1^ Department of Surgery Division of Surgical Oncology The Ohio State University Wexner Medical Center and James Comprehensive Cancer Center Columbus Ohio USA; ^2^ Department of Surgery University of Verona Verona Italy; ^3^ Department of Surgery San Raffaele Hospital Milan Italy; ^4^ Department of Surgery University of Virginia School of Medicine Charlottesville Virginia USA; ^5^ Department of Surgery Curry Cabral Hospital Lisbon Portugal; ^6^ Department of Surgery University of Ottawa Ottawa Canada; ^7^ Department of Surgery Fundeni Clinical Institute Bucharest Romania; ^8^ Department of Surgery Northwell Health Long Island New York USA; ^9^ Department of Surgery Keio University Tokyo Japan; ^10^ Department of Surgery Stanford University School of Medicine Stanford California USA; ^11^ Department of Surgery Emory University School of Medicine Atlanta Georgia USA; ^12^ Department of Surgery Westmead Hospital Sydney Australia; ^13^ Department of Surgery School of Medicine The University of Sydney Sydney Australia; ^14^ Department of Surgery University of Colorado Denver Colorado USA; ^15^ Department of Surgery Eastern Hepatobiliary Surgery Hospital Shanghai China; ^16^ Department of Surgery AP‐HP Beaujon Hospital Clichy France; ^17^ Department of Surgery Erasmus University Medical Centre Rotterdam the Netherlands; ^18^ Department of Surgery Yokohama City University School of Medicine Yokohama Japan

**Keywords:** inflammatory biomarker, liver cancer, lymphocyte‐C‐reactive protein ratio, overall survival, postoperative complications, recurrence‐free survival

## Abstract

**Objective:**

We sought to characterize the prognostic value of lymphocyte‐C‐reactive protein ratio (LCR) among patients undergoing liver resection (LR) for hepatocellular carcinoma (HCC) and intrahepatic cholangiocarcinoma (ICC).

**Methods:**

Patients who underwent curative‐intent LR for HCC and ICC between 2000 and 2023 were identified from a multiinstitutional database. The prognostic value of nine different inflammatory markers were evaluated relative to short‐ (i.e., postoperative morbidity) and long‐term (recurrence‐free survival [RFS] and overall survival [OS]) outcomes.

**Results:**

Among 715 patients, 499 (69.8%) and 216 (30.2%) individuals were included in the derivation and validation cohorts, respectively. Patients with advanced disease and poor tumor biology had lower median levels of LCR. An optimal LCR cutoff threshold of 6100 was identified in the derivation cohort. LCR demonstrated the highest accuracy to predict RFS and OS, with areas under the ROC curve of 0.724 and 0.716, respectively. After adjusting for relevant clinicodemographic factors, lower LCR remained associated with higher odds of postoperative complications (OR: 1.98 [95% CI: 1.27–3.10] and *p* = 0.003) and particularly, infectious complications (OR: 2.80 [95% CI: 1.57–5.01] and *p* < 0.001). A lower LCR was independently associated with worse RFS (HR: 2.43 [95% CI: 1.41–3.83] and *p* = 0.002) and OS (HR: 2.95 [95% CI: 2.10–4.16] and *p* < 0.001). The prognostic ability of LCR for short‐ and long‐term outcomes performed well in an independent validation cohort.

**Conclusion:**

LCR was strongly associated with risk of postoperative morbidity as well as worse RFS and OS among patients undergoing LR for HCC and ICC. Preoperative LCR assessment can aid surgeons in the preoperative risk‐stratification of patients undergoing surgery for primary liver cancer.

## Graphical abstract

We sought to characterize the prognostic value of lymphocyte‐C‐reactive protein ratio (LCR) among patients undergoing liver resection (LR) for hepatocellular carcinoma (HCC) and intrahepatic cholangiocarcinoma (ICC). Patients who underwent curative‐intent LR for HCC and ICC between 2000 and 2023 were identified from a multi‐institutional database. The prognostic value of nine different inflammatory markers were evaluated relative to short‐ (i.e., postoperative morbidity) and long‐term (recurrence‐free survival [RFS] and overall survival [OS]) outcomes.

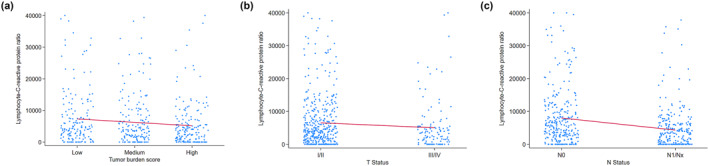

## 1 Introduction

Despite the increasing availability of treatment options and improvements in patient selection, primary liver cancer remains a therapeutic challenge [1, 2, 3]. Liver resection (LR) is the cornerstone of curative‐intent treatment for patients with primary liver cancer including hepatocellular carcinoma (HCC) and intrahepatic cholangiocarcinoma (ICC) [4]. Hwever, long‐term prognosis after resection remains poor due to a high incidence of recurrence up to 60%–70% within 5 years following surgery [5, 6, 7]. Similarly, despite improvements in perioperative care and operative techniques, postoperative morbidity remains high ranging from 20% to 56% [8, 9, 10]. To improve perioperative and long‐term survival outcomes, integrating novel biomarkers is crucial for predicting postoperative morbidity and disease recurrence, which may enhance perioperative decision‐making and postoperative care of patients.

Systemic inflammation, through host‐tumor interactions, is considered one of the hallmarks of cancer [11]. In the tumor microenvironment, persistent inflammation has been demonstrated to contribute to the proliferation and survival of malignant cells, angiogenesis, and metastasis in various cancers [11, 12, 13]. Preoperative systemic inflammatory response is also an independent factor associated with postoperative complications among patients undergoing cancer surgery [14]. Building on this understanding, a growing body of evidence has highlighted the potential of systemic inflammatory markers as prognostic biomarkers across multiple cancers including HCC and ICC [15, 16, 17, 18, 19]. Similarly, ratios combining different biomarkers, including the C‐reactive protein (CRP)‐albumin ratio (CAR), neutrophil‐to‐lymphocyte ratio (NLR), and platelet‐to‐lymphocyte ratio (PLR), have been proposed in an attempt to improve cancer prognostication. For instance, Okugawa Y et al. reported that preoperative lymphocyte–CRP ratio (LCR) was a promising biomarker to predict postoperative morbidity, recurrence, and long‐term survival among patients undergoing surgery for colorectal cancer (CRC) [20]. LCR capitalizes on the balance between lymphocytes, which reflect the body's immune‐nutrition status and immune response to cancer, and CRP, an indicator of systemic inflammation driven by tumor activity [19, 21, 22]. This combined immune‐inflammation approach makes LCR a promising prognostic biomarker that may perform better than other inflammatory biomarkers to predict oncological outcomes among patients with CRC and gastric cancer [20, 23].

Therefore, the objective of the current study was to characterize the prognostic value of preoperative LCR relative to other combinations of inflammatory biomarkers to predict postoperative complications, as well as long‐term outcomes including recurrence‐free (RFS) and overall survival (OS), among patients undergoing resection of primary liver cancer. Using a multi‐institutional cohort of patients with HCC and ICC, we sought to characterize an optimal cutoff for LCR that optimally stratified oncological outcomes. To ensure the reliability and generalizability of our findings, the performance of the identified LCR cutoff was validated in an independent external cohort.

## 2 Materials and Methods

### 2.1 Study Population and Exclusion Criteria

An international multi‐institutional database comprised of data from 18 major hepatobiliary institutions worldwide was queried to identify patients who underwent curative‐intent LR for HCC and ICC between 2000 and 2023. Exclusion criteria included individuals with metastatic disease (M1), grossly positive surgical margins (R2), patients who had a palliative resection, and individuals with missing data on preoperative lymphocyte count, CRP, or follow‐up information. Although HCC and ICC have distinct underlying biology, both are primarily treated with curative‐intent LR and share common perioperative risk profiles. Given the focus on evaluating the systemic inflammatory response as a prognostic factor across surgically treated primary liver cancers, patients with HCC and ICC were analyzed together. However, all multivariable models were adjusted for diagnosis, and subgroup analyses were performed to validate findings within each tumor type. The study received approval from the Institutional Review Boards of all participating institutions and followed the Transparent Reporting of a multivariable prediction model for Individual Prognosis or Diagnosis (TRIPOD) guidelines [24].

### 2.2 Patient Characteristics, Definitions, and Outcomes

Data on patient demographics, including age, sex, race/ethnicity, American Society of Anesthesiologists (ASA) physical status classification, and liver cirrhosis, were extracted. Clinicopathological factors included radiologic tumor size and number, lymph node status, tumor marker levels (alpha‐fetoprotein [AFP] for HCC and carbohydrate antigen 19‐9 [CA 19‐9] for ICC), tumor histological grade, perineural invasion, and resection margin status (R0/R1). Similarly, data on operative factors, such as surgical approach (open or minimally invasive surgery [MIS]), estimated intraoperative blood loss (mL), and duration of the operation (mins), were obtained. Additionally, data on lymph node dissection, biliary reconstruction, vascular resection, and receipt of adjuvant chemotherapy were also extracted for patients with ICC.

The tumor burden score (TBS) was calculated based on the radiologic maximum tumor diameter (cm) and the number of liver lesions using the formula: TBS^2^ = (maximum tumor diameter)^2^ + (number of lesions)^2^ [25]. Patients were categorized into three tertiles—low, medium, and high—based on the distribution of tumor marker levels. Similarly, the albumin–bilirubin (ALBI) score was determined using serum albumin (g/L) and total bilirubin (μmol/L) levels, with ALBI grades assigned according to predefined thresholds [26]. The American Joint Committee on Cancer (AJCC) tumor (T) and lymph node (N) status were defined according to the 8th edition of AJCC guidelines [27].

Data on preoperative levels related to five inflammation‐related factors were collected, including upregulated markers (neutrophils, platelets, and CRP) and downregulated markers (lymphocytes and albumin). These factors were chosen a priori as representative of inflammation‐based prognostic markers (NLR, PLR, and CAR), which had been previously proposed to predict survival outcomes among patients undergoing resection for a malignant indication [23, 28, 29, 30]. Nine different combinations of these five factors, including both ratios and multiples (Supporting Information S1: Table S1), were examined relative to prediction of RFS among patients undergoing LR for HCC and ICC.

Short‐term postoperative complications were defined as occurring within 30 days of LR. Postoperative infectious complications included wound infection (superficial or deep), intra‐abdominal abscess, cholangitis, respiratory tract infection, urinary tract infection, sepsis, or any distant infection. Recurrence was identified either through suspicious imaging findings or by histological confirmation of a tumor relapse. Long‐term outcomes of interest included RFS and OS, with RFS defined as the time from LR to cancer recurrence or death, and OS as the time from LR to death or last follow‐up.

### 2.3 Statistical Analyses

The analytic cohort was split into a derivation cohort, which included patients from 12 major Eastern and Western hepatobiliary institutions, and a validation cohort comprised of patients from six different hospitals.

Receiver operating characteristic (ROC) curves were generated for each inflammatory biomarker to predict RFS, and the area under the ROC curve (AUROC) was calculated to evaluate the discriminatory power of each biomarker [31]. Youden's index from the ROC curves was utilized to determine the optimal cutoff value for each biomarker [32]. Logistic regression analysis was employed to develop a multivariable model to predict postoperative complications, with a separate model that specifically examined postoperative infectious complications. Outcomes were expressed as linear predictors to assess correlation strength; odds ratios (ORs) with 95% confidence intervals (CIs) were reported. Univariable and multivariable Cox proportional hazards models were developed to predict long‐term RFS and OS, with results reported as hazards ratios (HRs) with 95% CI. In addition to LCR, clinicopathologic variables previously recognized as confounding factors that may influence postoperative morbidity and prognosis among patients with liver cancer were considered in the models. Factors were included in the multivariable models through a backward selection method, with a significance threshold set at *p* < 0.050. Kaplan–Meier survival curves for RFS and OS were plotted with stratification based on the LCR cutoff, and the log‐rank test was applied to compare the curves.

Descriptive statistics for categorical variables were reported as frequencies and percentages (%); comparisons were conducted using either the chi‐squared test or Fisher’s exact test. Continuous variables were expressed as median values with interquartile ranges (IQRs), with comparisons made using the Mann–Whitney *U* test. The multiple imputation by chained equations (MICE) technique was employed to impute missing values [33]. All statistical tests were two‐tailed, with the level of statistical significance set at *p* < 0.05. All statistical analyses were conducted using Python version 3.11 in Visual Studio Code version 1.84.2 and Stata version 18.0 (StataCorp).

## 3 Results

### 3.1 Patient Characteristics

A total of 715 patients who underwent curative‐intent LR (HCC: *n* = 448, 62.7% and ICC: *n* = 267, 37.3%) met inclusion criteria and were included in the analytic cohort (Table 1). Median patient age was 65.0 years (IQR: 56.0–73.0), 513 (71.7%) patients were male, and 202 (28.3%) individuals had an ASA class > 2. Median TBS was 5.1 (IQR: 3.4–7.9) and 13.0% (*n* = 93) of patients had suspicious or metastatic lymph nodes on the preoperative radiologic assessment. Median preoperative CRP, lymphocyte, and platelet count were 0.28 mg/dL (IQR: 0.02–0.84), 1460.0 per μL (IQR: 1090.3–1960.0), and 188.0 × 10^3^ per μL (IQR: 137.0–247.0), respectively; median LCR was 6072 (IQR: 16.9–54,286).

**TABLE 1:** Clinicodemographic characteristics and outcomes in the analytic cohort and comparison between patients in the derivation and validation cohorts.

Variables	All patients (*n* = 715)	Derivation cohort (*n* = 499, 69.8%)	Validation cohort (*n* = 216, 30.2%)	*p* value
Diagnosis				
HCC	448 (62.7%)	314 (62.9%)	134 (62.0%)	0.822
ICC	267 (37.3%)	185 (37.1%)	82 (38.0%)	
Age (years)	65.0 (56.0–73.0)	65.0 (57.0–73.0)	63.0 (55.0–71.0)	0.056
Sex				
Male	513 (71.7%)	361 (72.3%)	152 (70.4%)	0.590
Female	202 (28.3%)	138 (27.7%)	64 (29.6%)	
Race				
White	336 (47.0%)	235 (47.1%)	101 (46.8%)	0.318
Black	5 (0.7%)	2 (0.4%)	3 (1.4%)	
Asian	368 (51.5%)	256 (51.3%)	112 (51.9%)	
Hispanic	3 (0.4%)	3 (0.6%)	0 (0.0%)	
Other	3 (0.4%)	3 (0.6%)	0 (0.0%)	
ASA class > 2	202 (28.3%)	140 (28.1%)	62 (28.7%)	0.860
Cirrhosis	239 (33.4%)	164 (32.9%)	75 (34.7%)	0.629
BMI (kg/m^2^)	24.7 (22.2–27.4)	24.8 (22.3–27.9)	24.4 (22.2–26.3)	0.070
Tumor marker				
Low	239 (33.4%)	167 (33.5%)	72 (33.3%)	0.727
Medium	238 (33.3%)	162 (32.5%)	76 (35.2%)	
High	238 (33.3%)	170 (34.1%)	68 (31.5%)	
TBS	5.1 (3.4–7.9)	5.1 (3.4–8.0)	5.0 (3.3–7.6)	0.675
Radiologic lymph nodes status				
Negative	622 (87.0%)	434 (87.0%)	188 (87.0%)	0.982
Suspicious/Positive	93 (13.0%)	65 (13.0%)	28 (13.0%)	
Neoadjuvant chemotherapy	27 (3.8%)	23 (4.6%)	4 (1.9%)	0.046
Neutrophil count (per μL)	3700.0 (2760.0–5010.0)	3672.9 (2734.2–4835.4)	3726.7 (2775.7–5361.7)	0.430
Lymphocyte count (per μL)	1460.0 (1090.3–1960.0)	1490.8 (1090.3–2000.0)	1400.1 (1092.2–1894.1)	0.533
Platelet count (*10^3^ per μL)	188.0 (137.0–247.0)	190.0 (140.0–250.0)	180.5 (134.5–230.0)	0.362
Albumin (g/dL)	4.2 (3.9–4.4)	4.1 (3.8–4.4)	4.2 (3.9–4.4)	0.187
ALBI grade				
1	571 (79.9%)	392 (78.6%)	179 (82.9%)	0.187
2/3	144 (20.1%)	107 (21.4%)	37 (17.1%)	
CRP (mg/dL)	0.28 (0.02–0.84)	0.26 (0.02–0.8)	0.30 (0.04–1.00)	0.990
LCR	6072 (16.9–54286)	6460 (18.8–59818)	4838 (12.1–36281)	0.382
Surgical approach				
Open	539 (75.4%)	379 (76.0%)	160 (74.1%)	0.592
MIS	176 (24.6%)	120 (24.0%)	56 (25.9%)	
Operative blood loss (mL)	340.0 (150.0–746.6)	400.0 (150.0–761.4)	300.0 (100.0–714.5)	0.764
Operative time (minutes)	276.0 (180.0–360.0)	286.0 (182.0–376.2)	262.5 (174.7–330.6)	0.021
Postoperative complications	228 (31.8%)	161 (32.2%)	67 (31.0%)	0.823
Infectious complications	116 (16.2%)	77 (15.5%)	39 (18.1%)	0.038
Major vascular invasion	94 (13.1%)	68 (13.6%)	26 (12.0%)	0.563
Perineural invasion	155 (21.7%)	115 (23.0%)	40 (18.5%)	0.028
Tumor grade				
Well/Moderately differentiated	432 (60.4%)	299 (59.9%)	133 (61.6%)	0.678
Poorly/Undifferentiated	283 (39.6%)	200 (40.1%)	83 (38.4%)	
Location				
Unilobar	578 (81.1%)	407 (81.9%)	171 (79.2%)	0.393
Bilobar	135 (18.9%)	90 (18.1%)	45 (20.8%)	
Resection margin				
R0	625 (87.4%)	432 (86.6%)	193 (89.4%)	0.304
R1	90 (12.6%)	67 (13.4%)	23 (10.6%)	
T status				
I/II	584 (81.7%)	406 (81.4%)	178 (82.4%)	0.740
III/IV	131 (18.3%)	93 (18.6%)	38 (17.6%)	
N status				
N0	415 (58.0%)	284 (56.9%)	131 (60.6%)	0.649
N1	57 (8.0%)	41 (8.2%)	16 (7.4%)	
Nx	243 (34.0%)	174 (34.9%)	69 (31.9%)	
Adjuvant chemotherapy	76 (10.6%)	62 (12.4%)	14 (6.5%)	0.018

Abbreviations: ALBI, albumin–bilirubin; ASA, American Society of Anesthesiologists; BMI, body mass index; CRP, C‐reactive protein; HCC, hepatocellular carcinoma; ICC, intrahepatic cholangiocarcinoma; LCR, lymphocyte‐C‐reactive protein ratio; MIS, minimally invasive surgery; TBS, tumor burden score.

At the time of surgery, most patients (*n* = 539, 75.4%) underwent LR via an open approach. On final pathology, 584 (81.7%) patients had either T1 or T2 disease; 432 (60.4%) patients had well‐ or moderately differentiated tumors and an R0 resection margin was achieved in 87.4% (*n* = 625) of operative cases. Among patients with ICC, 46.8% (*n* = 125) underwent lymph node dissection, 17.1% (*n* = 46) had vascular resection, 19.8% (*n* = 53) underwent biliary reconstruction, and 33.4% (*n* = 89) received adjuvant chemotherapy. Overall, roughly one‐third of patients (*n* = 228, 31.8%) experienced postoperative morbidity; 116 (16.2%) patients had an infectious complication within 30 days of surgery.

**FIGURE 1:**
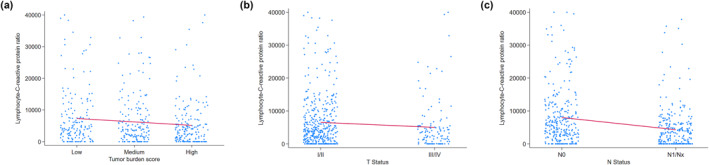
(a) Preoperative lymphocyte‐C‐reactive protein ratio stratified by tumor burden score (low vs. medium vs. high). (b) Preoperative lymphocyte‐C‐reactive protein ratio stratified by T status (T1/T2 vs. T3/T4). (c) Preoperative lymphocyte‐C‐reactive protein ratio stratified by N status (N0 vs. N1/Nx).

The derivation cohort consisted of 499 (69.8%) patients, whereas the validation cohort consisted of 216 (30.2%) patients. Both cohorts were largely comparable across clinicodemographic variables and perioperative outcomes; however, there were differences in operative time, perineural invasion, infectious complications, and receipt of neoadjuvant and adjuvant chemotherapy (all *p* < 0.05) (Table 1). In examining the derivation cohort, lower preoperative LCR correlated with worse clinicopathological factors and advanced tumor biology (Table 2). Patients with high TBS (high: 4443.7 vs. medium: 6507.0 and low: 7707.0 and *p* = 0.004), major vascular invasion (present: 2694.0 vs. absent: 7390.0 and *p* < 0.001), poorly or undifferentiated tumor grade (poorly/undifferentiated: 5993.3 vs. well/moderately differentiated: 9032.6 and *p* = 0.014), perineural invasion (present: 3961.5 vs. absent: 8157.8 and *p* < 0.001), T3 or T4 tumors (T3/T4: 2575.1 vs. T1/T2: 8157.8 and *p* < 0.001), and lymph node metastasis (N1/Nx: 4964.8 vs. N0: 8531.3 and *p* < 0.001) were more likely to have lower preoperative LCR (Figure 1a–c).

**TABLE 2:** Comparison of lymphocyte‐C‐reactive protein ratio across clinicodemographic variables in the derivation cohort.

Variables	Number (%)	Median LCR	IQR	*p* value
Age				
< 65 years	233 (46.7%)	6266.7	56794.4	0.906
≥ 65 years	266 (53.3%)	6789.1	57076.1	
Sex				
Male	361 (72.3%)	5993.3	54267.5	0.196
Female	138 (27.7%)	8406.5	70924.4	
ASA class				
≤ 2	359 (71.9%)	6897.8	74151.8	0.484
> 2	140 (28.1%)	5930.7	26821.1	
Tumor marker				
Low	167 (33.5%)	6812.1	60472.1	0.956
Medium	162 (32.5%)	6047.16	43190.5	
High	170 (34.0%)	7619.8	60491.1	
TBS				
Low	167 (33.5%)	7707.0	12615.0	0.004
Medium	162 (32.5%)	6507.0	48332.4	
High	170 (34.0%)	4443.7	13595.7	
ALBI grade				
1	392 (78.6%)	10904.5	10924.3	0.271
2/3	107 (21.4%)	6205.9	42608.8	
Major vascular invasion				
Absent	431 (86.4%)	7390.0	87301.0	< 0.001
Present	68 (13.6%)	2694.0	28940.0	
Tumor grade				
Well/Moderately differentiated	299 (59.9%)	9032.6	12108.8	0.014
Poorly/Undifferentiated	200 (40.1%)	5993.3	24278.1	
Perineural invasion				
Absent	384 (77.0%)	8157.8	10706.9	< 0.001
Present	115 (23.0%)	3961.5	7418.1	
Resection margin				
R0	432 (86.6%)	7143.5	78617.8	0.004
R1	67 (13.4%)	3933.3	8579.1	
T status				
I/II	406 (81.4%)	8157.8	97529.6	< 0.001
III/IV	93 (18.6%)	2575.1	7767.1	
N status				
N0	283 (56.7%)	8531.3	11942.6	< 0.001
N1/Nx	216 (43.3%)	4964.8	27037.9	

Abbreviations: ALBI, albumin–bilirubin; ASA, American Society of Anesthesiologists; IQR, interquartile range; LCR, lymphocyte‐C‐reactive protein ratio; TBS, tumor burden score.

### 3.2 Prognostic Impact of Inflammatory Biomarkers

**FIGURE 2:**
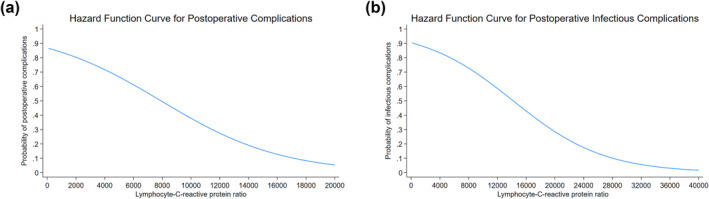
(a) Kaplan–Meier curves for recurrence‐free survival in the derivation cohort, stratified by lymphocyte‐C‐reactive protein ratio: low versus high. (b) Kaplan–Meier curves for overall survival in the derivation cohort, stratified by lymphocyte‐C‐reactive protein ratio: low versus high.

A low LCR was independently associated with higher odds of a postoperative complication (OR: 1.98 [95% CI: 1.27–3.10] and *p* = 0.003) including postoperative infectious complications (OR: 2.80 [95% CI:1.57–5.01] and *p* < 0.001) (Table 3). Logistic regression analyses adjusted for age, sex, diagnosis, ASA classification, liver cirrhosis, ALBI grade, tumor histological grade, T status, N status, surgical approach, operation time, and intraoperative blood loss confirmed this association (Supporting Information S1: Tables S2 and S3). Hazard function curves demonstrated a downward trend in the probability of any postoperative complications and postoperative infectious complications with increasing LCR (Figure 2a,b).

**TABLE 3:** Prognostic impact of low versus high lymphocyte‐C‐reactive protein ratio on short‐ and long‐term outcomes on multivariable regression analyses.

Outcomes	Derivation cohort		Validation cohort	
	OR/HR (95% CI)	*p* value	OR/HR (95% CI)	*p* value
Short‐term outcomes				
Any postoperative complication	1.98 (1.27–3.10)	0.003	2.08 (1.07–4.35)	0.039
Postoperative infectious complication	2.80 (1.57–5.01)	< 0.001	5.81 (2.28–7.36)	0.001
Long‐term outcomes				
Recurrence‐free survival	2.43 (1.41–3.83)	0.002	2.89 (1.39–5.57)	0.002
Overall survival	2.95 (2.10–4.16)	< 0.001	2.96 (1.15–4.42)	0.001

*Note:* The multivariable models for short‐term outcomes were adjusted for age, sex, diagnosis, American Society of Anesthesiologists classification, liver cirrhosis, albumin–bilirubin grade, tumor histological grade, T status, N status, surgical approach, operation time, and intraoperative blood loss. The multivariable models for long‐term outcomes were adjusted for age, sex, diagnosis, American Society of Anesthesiologists classification, liver cirrhosis, albumin–bilirubin grade, tumor marker, tumor grade, perineural invasion, tumor location, T status, N status, and surgical resection margin status.

Abbreviations: CI, confidence interval; HR, hazards ratio; OR, odds ratio.

**FIGURE 3:**
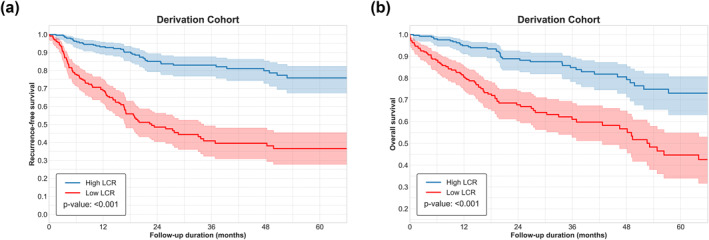
(a) Hazard function curve for probability of postoperative complications relative to lymphocyte‐C‐reactive protein ratio. (b) Hazard function curve for probability of postoperative infectious complications relative to lymphocyte‐C‐reactive protein ratio.

Patients with low LCR had a median RFS of 10.0 months (95% CI: 7.2–12.6 months) compared with a median RFS of 24.6 months (95% CI: 21.7–28.8 months) among patients with high LCR (Figure 3a). Similarly, median OS among patients with low and high LCR was 16.3 months (95% CI: 14.3–18.7 months) and 24.8 months (95% CI: 22.3–29.8 months), respectively (Figure 3b). On multivariable Cox regression analyses of the derivation cohort data, low LCR (< 6100) remained independently associated with worse RFS (HR: 2.43 [95% CI: 1.41–3.83] and *p* = 0.002) and OS (HR: 2.95 [95% CI: 2.10–4.16] and *p* < 0.001) (Table 3). These multivariable models were adjusted for potentially confounding clinicopathological factors, including age, sex, diagnosis, ASA classification, liver cirrhosis, ALBI grade, tumor marker, tumor grade, perineural invasion, tumor location, T status, N status, and resection margin status (Supporting Information S1: Tables S4 and S5). On sensitivity analysis, LCR remained associated with worse RFS and OS among patients with both HCC and ICC (Supporting Information S1: Tables S6–S9).

ROC curve analysis was conducted in the derivation cohort to assess the prognostic value of nine different preoperative inflammatory biomarkers, including LCR, to predict RFS (Supporting Information S1: Figure S1). Of note, LCR had the highest accuracy to predict worse RFS among the different inflammation‐related factors, achieving an AUROC of 0.724 (95% CI: 0.675–0.768) (*p* < 0.001) (Table 4). The optimal cutoff of 6100 for LCR demonstrated a sensitivity of 61.6% and a specificity of 72.8% to predict poor RFS. Similar results were observed for OS. Specifically, among the different inflammation‐related markers, LCR had the highest discrimination with an AUROC of 0.716 (95% CI: 0.662–0.769) (*p* < 0.001) and a sensitivity and specificity of 46.7% and 87.6%, respectively (Supporting Information S1: Figure S2). Of note, LCR AUROC values for both DFS and OS were higher than other inflammatory biomarkers (Supporting Information S1: Table S10).

**TABLE 4:** Receiver operating characteristic curve analysis for recurrence‐free and overall survival in the derivation cohort.

Inflammatory markers	ROC curve analysis for recurrence‐free survival				ROC curve analysis for overall survival			
	AUROC	Sensitivity	Specificity	*p* value	AUROC	Sensitivity	Specificity	*p* value
Lymphocyte–CRP ratio	0.724 (0.675–0.768)	61.6%	72.8%	< 0.001	0.716 (0.662–0.769)	46.7%	87.6%	< 0.001
Neutrophil‐to‐lymphocyte ratio	0.666 (0.615–0.718)	42.2%	87.2%	0.001	0.601 (0.537–0.663)	43.3%	79.4%	0.035
Neutrophil × platelet count	0.623 (0.562–0.683)	70.2%	60.0%	0.020	0.623 (0.562–0.683)	70.2%	60.0%	0.020
Neutrophil count × CRP	0.653 (0.599–0.701)	63.3%	61.6%	< 0.001	0.669 (0.614–0.721)	47.5%	80.2%	< 0.001
CRP/Albumin ratio	0.681 (0.626–0.728)	57.8%	71.3%	< 0.001	0.703 (0.653–0.759)	84.7%	49.2%	< 0.001
Lymphocyte count × albumin	0.637 (0.585–0.689)	52.8%	74.1%	0.002	0.560 (0.502–0.623)	41.7%	70.0%	0.091
Platelet count × CRP	0.661 (0.604–0.712)	61.9%	64.2%	< 0.001	0.665 (0.608–0.718)	65.0%	64.1%	< 0.001
Platelet–albumin ratio	0.533 (0.477–0.590)	48.3%	60.5%	0.251	0.607 (0.545–0.666)	35.8%	84.7%	0.005
Platelet‐to‐lymphocyte ratio	0.640 (0.587–0.696)	37.4%	88.9%	0.004	0.592 (0.526–0.655)	32.5%	87.6%	0.038

Abbreviations: AUROC, area under the receiver operating characteristic curve; CRP, C‐reactive protein.

### 3.3 External Validation

The performance of LCR to predict short‐ and long‐term outcomes among patients with primary liver cancer was externally validated in a separate cohort of patients (*n* = 216). Consistent with findings in the derivation cohort, patients with advanced disease were more likely to have lower preoperative LCR (Supporting Information S1: Table S11). Using the cutoff value of 6,100, which was identified in the derivation cohort, a low LCR was associated with a higher incidence of any postoperative complication as well as postoperative infectious complications in the validation cohort (Supporting Information S1: Tables S12 and S13). In addition, low LCR was associated with worse RFS and OS in the validation cohort (Supporting Information S1: Tables S14 and S15) (Supporting Information S1: Figure S3a,b).

## 4 Discussion

Systemic inflammation and immune dysregulation play a crucial role in cancer development and progression [11, 34]. Cancer cells, along with surrounding stromal and inflammatory cells, engage in reciprocal interactions that establish an inflammatory tumor microenvironment supporting tumor growth and metastasis [35]. Similarly, immune dysfunction characterized by perioperative lymphopenia has been linked to cancer progression and a poor prognosis among patients with gastric, pancreatic, and breast cancer [36, 37]. As such, a number of research studies have assessed the effectiveness of various systemic inflammation biomarkers and immune dysfunction to predict prognosis among patients with cancer [15, 16, 17, 18, 19]. Nevertheless, the most effective combination of systemic inflammatory markers to predict both short‐ and long‐term postoperative outcomes among patients undergoing surgery for primary liver cancer had not been investigated. Therefore, the current study was important because we specifically evaluated and compared the prognostic value of nine different combinations of inflammatory biomarkers among patients who underwent curative‐intent surgery for HCC and ICC. Of note, a low LCR was independently associated with a higher incidence of postoperative complications, including infectious complications. LCR also had the highest accuracy to predict worse RFS, achieving an AUROC of 0.724 (95% CI: 0.675–0.768), with a sensitivity of 61.6% and a specificity of 72.8%. Interestingly, a lower LCR was associated with higher TBS, more advanced T and N stages and poor tumor histopathology—likely accounting for the worse prognosis. These findings were validated in the external cohort. Although HCC and ICC have distinct etiologies and natural histories, the impact of systemic inflammation on postoperative outcomes is a common biological mechanism shared across both malignancies. Therefore, for the purposes of analysis, both primary liver cancer diseases were combined to enhance cohort size and statistical power; however, we did adjust for diagnosis type in all multivariable models and confirmed the results in disease‐specific stratified analyses. The consistent association between low LCR and poor RFS and OS in both HCC and ICC strengthened the generalizability of LCR as a prognostic biomarker among patients undergoing liver resection for primary liver cancers. As such, use of LCR in the clinical setting may provide an easy‐to‐use means to help stratify patients with primary liver cancer relative to short‐ and long‐term outcomes.

Both local immune response and systemic inflammation, as well their complex interplay, have an important role in tumor progression and survival among patients with cancer [38]. Locally, the tumor microenvironment is characterized by cytokines, chemokines, and immune cells that engage in continuous intracellular communication, promoting tumor growth, angioinvasion, and metastasis [39]. Specifically, in the liver, in vivo studies have demonstrated that smoldering hepatic inflammation promotes carcinogenesis, with tumor necrosis factor (TNF) secreted by Kupffer cells driving tumorigenesis via JNK signaling under conditions of oxidative stress [40, 41, 42]. Interleukin‐6 (IL‐6) is another key factor in inflammation‐induced hepatocarcinogenesis, exacerbating oxidative stress, DNA damage, and gene mutations [43]. Additionally, tumor‐associated inflammation can lead to malnutrition, cachexia, and frailty, which impair patient performance status and surgical fitness, thereby contributing to a worse prognosis [44, 45]. Although existing studies have examined the prognostic value of LCR for HCC and ICC [46, 47], the current study was unique in its comprehensive approach. Using a large multicenter cohort that incorporated patients from both major Eastern and Western hospitals, we were able to utilize both a derivation and a separate validation cohort to test the generalizability of the findings. Beyond oncological and survival outcomes, we also specifically examined short‐term outcomes including postoperative and infectious complications. An optimal LCR cutoff was established that simplified clinical application and compared the prognostic impact of LCR against eight other combinations of five different inflammatory markers, providing a broader understanding of its efficacy relative to established biomarkers. These aspects underscore the unique contributions of the current study to the field.

Interestingly, low LCR was independently associated with short‐term outcomes following surgery including postoperative morbidity and, in particular, a higher incidence of postoperative infectious complications (Figure 3a,b). There is an established link between systemic inflammation and postoperative morbidity [14, 48]. Using the modified Glasgow prognostic score to characterize preoperative systemic inflammatory status, Moyes et al. reported that systemic inflammation independently predicted postoperative complications among patients undergoing curative‐intent CRC resection [14]. Given that postoperative infections are often preventable, LCR may help identify patients at highest risk of infectious complications facilitating early intervention. The Centers for Disease Control and Prevention (CDC) guidelines recommend that antimicrobial prophylaxis following liver surgery should be discontinued within a 24 h period [49]. Although our findings suggest that a low LCR is associated with an increased risk of postoperative infections, caution should be employed in the direct application of these results to extend antimicrobial therapy. Decisions regarding antimicrobial use should be guided by comprehensive clinical assessments, adherence to antimicrobial stewardship principles, and corroborated by additional biomarkers. Of note, prevention and early treatment of postoperative complications is important for multiple reasons, including that postoperative infectious may be associated with worse long‐term outcomes [10, 50].

Although TNM staging is a well‐established method to evaluate prognosis among patients with HCC and ICC, variability in outcomes among similar patient groups underscores the need for more effective biomarkers to identify individuals at high risk for adverse long‐term oncological outcomes [51]. To this end, identifying an accurate means to evaluate systemic inflammation may help personalized treatment and surveillance strategies for patients with primary liver cancer. The Biomarkers Definitions Working Group at National Institute of Health defines an ideal prognostic biomarker as one that independently predicts a patient's prognosis beyond conventional classifications, predicts responses or adverse effects to treatments to enhance outcomes and quality of life, and is cost‐effective, readily available, and simplifies clinical decision‐making by objectively stratifying patients into different risk groups [52]. The inflammatory status of the body can be assessed through various biochemical and hematological markers commonly included in routine blood tests or through derived ratios such as NLR, PLR, and CAR [28, 29, 30]. Although these markers have been studied in relation to cancer risk and prognosis, results have been inconsistent and their applicability to patients with primary liver cancer had not been defined [28, 53, 54].

In the current study, among patients with HCC and ICC, LCR demonstrated the highest accuracy to predict worse RFS and OS compared with eight other inflammatory biomarkers (Table 4). In fact, low LCR was independently associated with over a 2‐fold increased risk of recurrence and death even after adjusting for clinicodemographic factors such as TBS, T and N status, and tumor grade (Table 3). Notably, this association remained consistent on sensitivity analyses stratified by cancer diagnosis and was validated in an independent cohort of patients with HCC and ICC. CRP, a pentraxin family protein released by hepatocytes in response to IL‐6, is a marker of increased cancer risk and worse prognosis [55]. The sensitivity, specificity, reproducibility, and cost‐effectiveness of CRP make it a valuable prognostic factor in the clinical setting [56]. To this point, Zhu et al. reported that CRP levels correlated with cancer incidence, including liver cancer, demonstrating a nonlinear pattern of increasing cancer risk with rising CRP concentrations [57]. In contrast, lymphocytes play a central role in cancer immunosurveillance, facilitating the immune system's ability to detect and eliminate malignant cells [58, 59]. A previous study by our own research group noted that preoperative lymphopenia was independently associated with higher risk of recurrence and mortality following curative‐intent resection of HCC [19]. Therefore, a low LCR, characterized by lymphopenia and elevated CRP levels, likely suggests a compromised immunological response and/or increased systemic inflammation among patients with cancer. Evaluating preoperative LCR may identify patients at high risk of poor outcomes and, therefore, may benefit from consideration of neo/adjuvant therapy as well as closer surveillance in the postoperative period.

The findings of the current study should be interpreted in the light of several limitations. Although offering valuable prognostic insights, LCR simplifies the complex interactions within the tumor microenvironment. As a reductionist surrogate of cancer‐associated inflammation, LCR captures systemic inflammation effectively, yet it does not encompass the full spectrum of tumor biology. Future research should integrate such biomarkers with broader biological data to enhance prognostic accuracy. The retrospective design introduced the potential of selection bias and limited the ability to control for all potential confounding variables. Although the multi‐institutional approach strengthened the study, differences in surgical techniques and postoperative care across centers may have influenced outcomes. The study also focused exclusively on patients undergoing LR for HCC and ICC; as such, the findings may not be generalizable to individuals undergoing ablation, transplantation, or nonsurgical treatments. To establish the reliability and applicability of LCR, larger prospective trials are needed to evaluate its prognostic potential to identify patients with liver cancer at high risk for adverse short‐ and long‐term postoperative outcomes.

In conclusion, LCR was as a robust prognostic marker for patients undergoing curative‐intent LR for HCC and ICC relative to short‐ and long‐term outcomes. Patients with a low preoperative LCR generally had tumors with worse disease‐specific characteristics. LCR was strongly associated with both RFS and OS, demonstrating superior predictive accuracy than other inflammatory‐based tools. LCR may facilitate more effective preoperative risk stratification of patients with primary liver cancer and help guide more patients‐specific tailored clinical decision‐making.

## Author Contributions

**Abdullah Altaf**: conceptualization, data curation, formal analysis, writing – original draft. **Andrea Baldo**: conceptualization, formal analysis, writing – review and editing. **Mujtaba Khalil**: conceptualization, writing – review and editing, data curation. **Zayed Rashid**: conceptualization, data curation, writing – review and editing. **Miho Akabane**: conceptualization, data curation, writing – review and editing. **Shahzaib Zindani**: conceptualization, writing – review and editing, data curation. **Azza Sarfraz**: conceptualization, data curation, writing – review and editing. **Andrea Ruzzenente**: conceptualization, data curation, supervision, writing – review and editing. **Luca Aldrighetti**: conceptualization, data curation, supervision, writing – review and editing. **Todd W. Bauer**: conceptualization, data curation, supervision, writing – review and editing. **Hugo P. Marques**: conceptualization, data curation, supervision, writing – review and editing. **Guillaume Martel**: supervision, writing – review and editing, conceptualization, data curation. **Irinel Popescu**: conceptualization, data curation, supervision, writing – review and editing. **Mathew J. Weiss**: conceptualization, data curation, supervision, writing – review and editing. **Minoru Kitago**: conceptualization, data curation, supervision, writing – review and editing. **George Poultsides**: conceptualization, data curation, supervision, writing – review and editing. **Shishir K. Maithel**: conceptualization, data curation, supervision, writing – review and editing. **Vincent Lam**: writing – review and editing, conceptualization, data curation, supervision. **Tom Hugh**: supervision, writing – review and editing, conceptualization, data curation. **Ana Gleisner**: conceptualization, data curation, supervision, writing – review and editing. **Feng Shen**: writing – review and editing, supervision, conceptualization, data curation. **Francois Cauchy**: conceptualization, data curation, supervision, writing – review and editing. **Bas G. Koerkamp**: writing – review and editing, supervision, conceptualization, data curation. **Itaru Endo**: conceptualization, data curation, supervision, writing – review and editing. **Timothy M. Pawlik**: conceptualization, data curation, writing – original draft, methodology, supervision, project administration, writing – review and editing, resources.

## Conflicts of Interest

The authors declare no conflicts of interest.

## Supporting information

Supporting Information S1

## Data Availability

The data that support the findings of this study are available from the corresponding author upon request. The data are not publicly available due to privacy or ethical restrictions.
