# Supplementary material for: Lymphocyte‐C‐Reactive Protein Ratio: Impact on Prognosis of Patients Following Resection of Primary Liver Cancer
Source: World J Surg. 2025 Jun 17;49(8):2195–206. doi: 10.1002/wjs.12675 (PMC12338384; doi:10.1002/wjs.12675)
Supplement: Supplementary file 1 — Supporting Information S1 [file WJS-49-2195-s001.docx]

**Supplementary Figures and Tables**

**Supplementary Figure 1.** Receiver operating characteristic (ROC) curve analysis to investigate the prognostic value of different inflammatory biomarkers including the Lymphocyte-C-reactive protein ratio to predict recurrence-free survival in the derivation cohort.


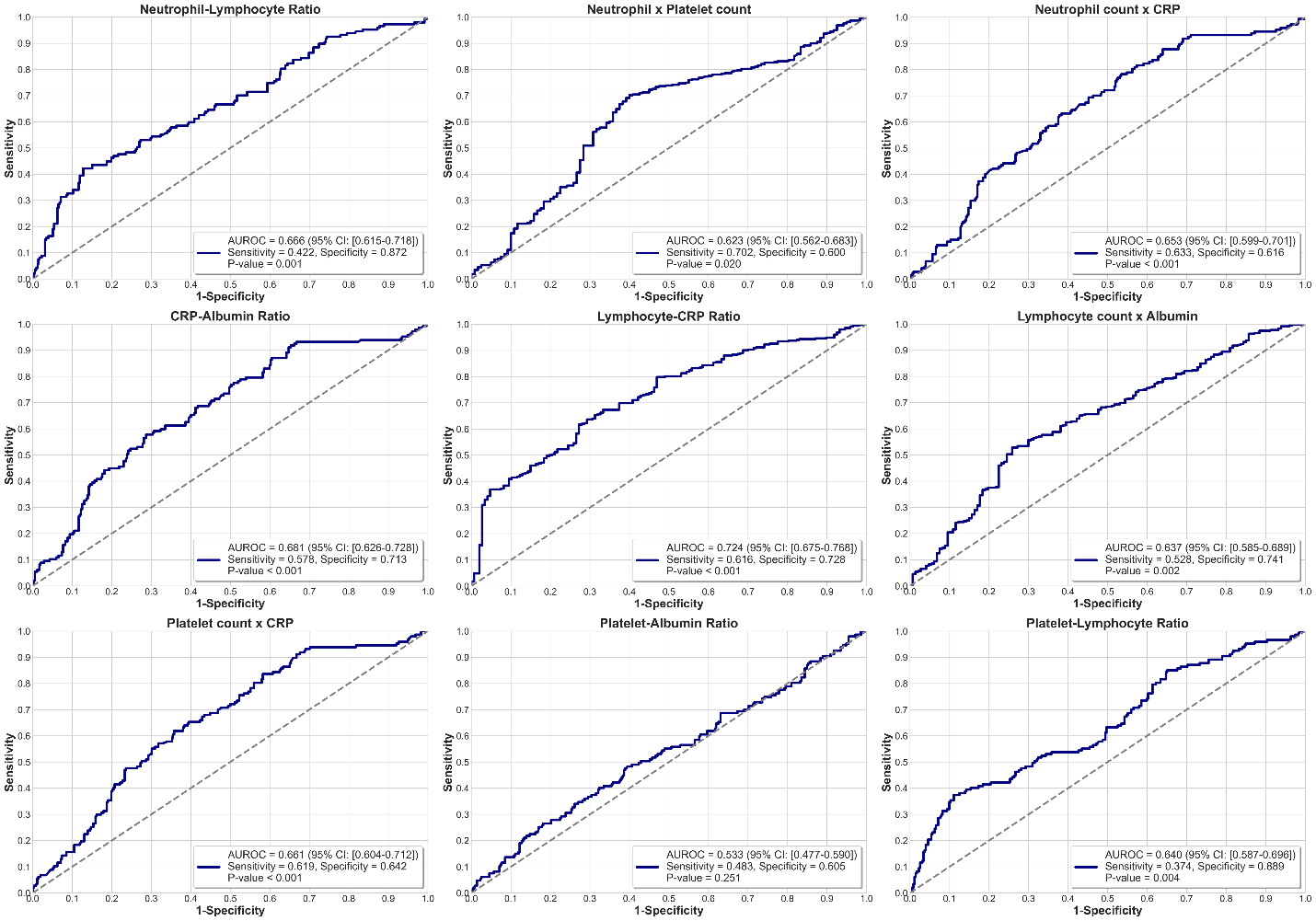


**Supplementary Figure 2.** Receiver operating characteristic (ROC) curve analysis to investigate the prognostic value of different inflammatory biomarkers including the Lymphocyte-C-reactive protein ratio to predict overall survival in the derivation cohort.


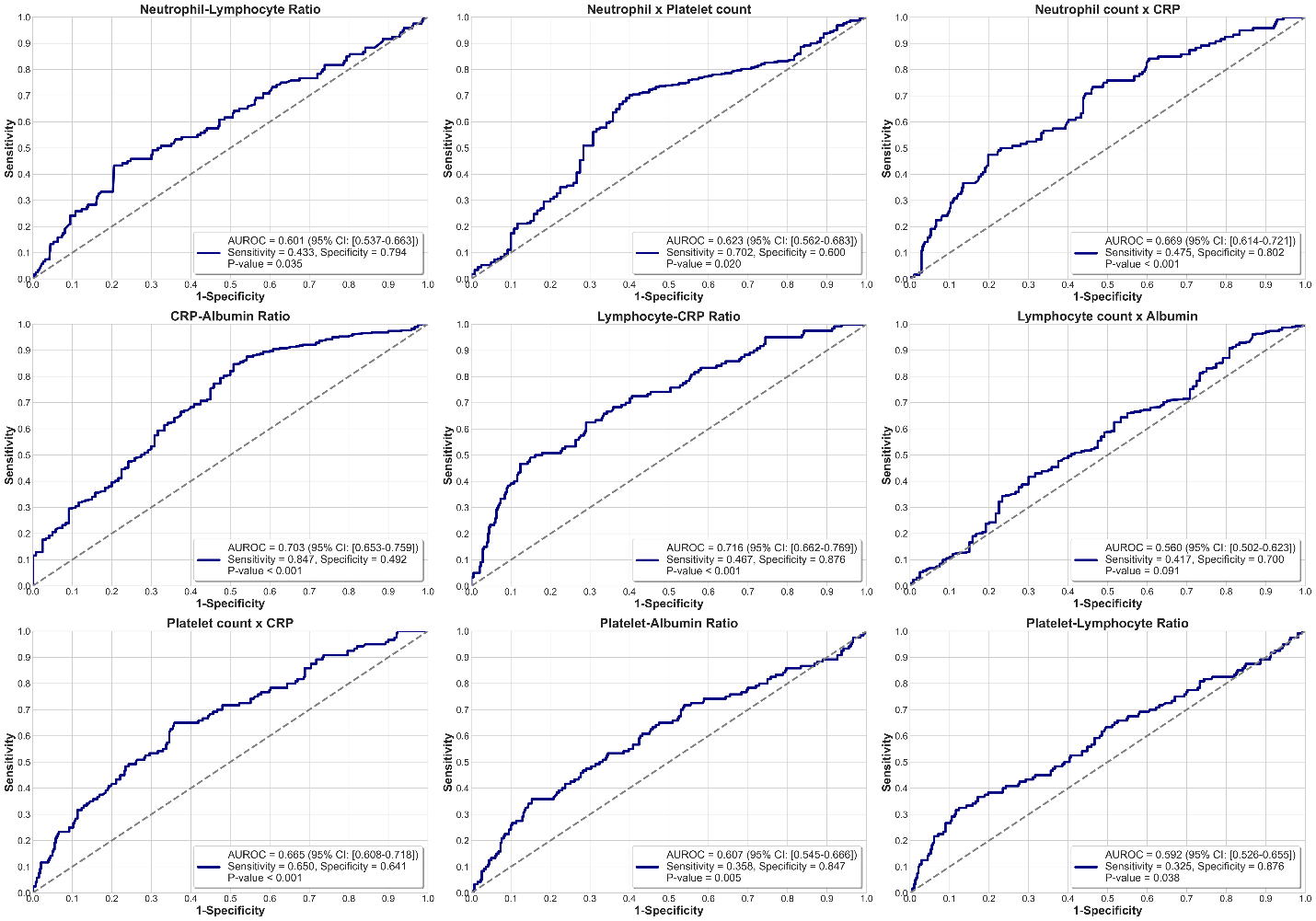


**Supplementary Figure 3a.** Kaplan–Meier curves for recurrence-free survival in the validation cohort, stratified by Lymphocyte-C-reactive protein ratio: low versus high.


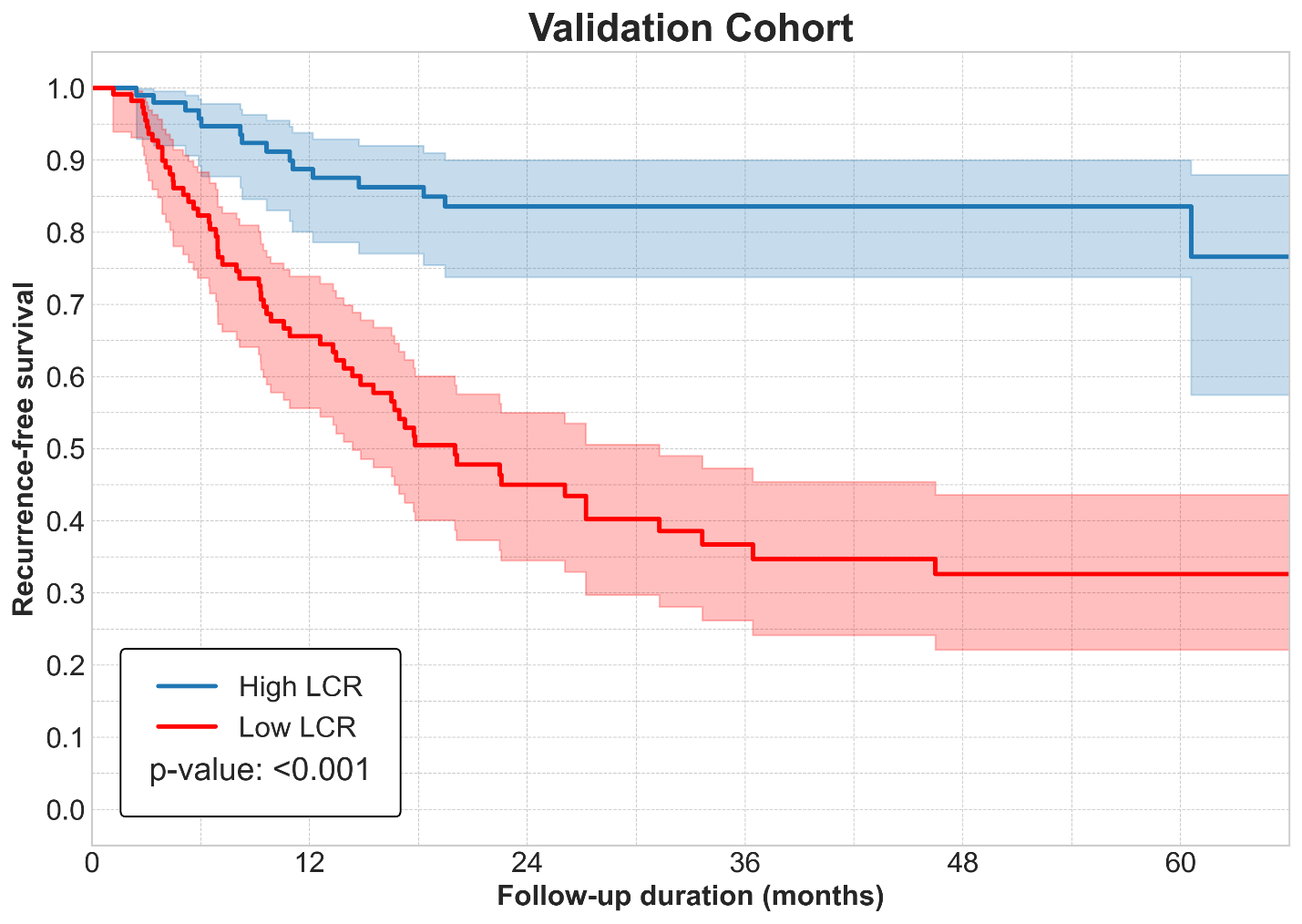


**Supplementary Figure 3b.** Kaplan–Meier curves for overall survival in the validation cohort, stratified by Lymphocyte-C-reactive protein ratio: low versus high.
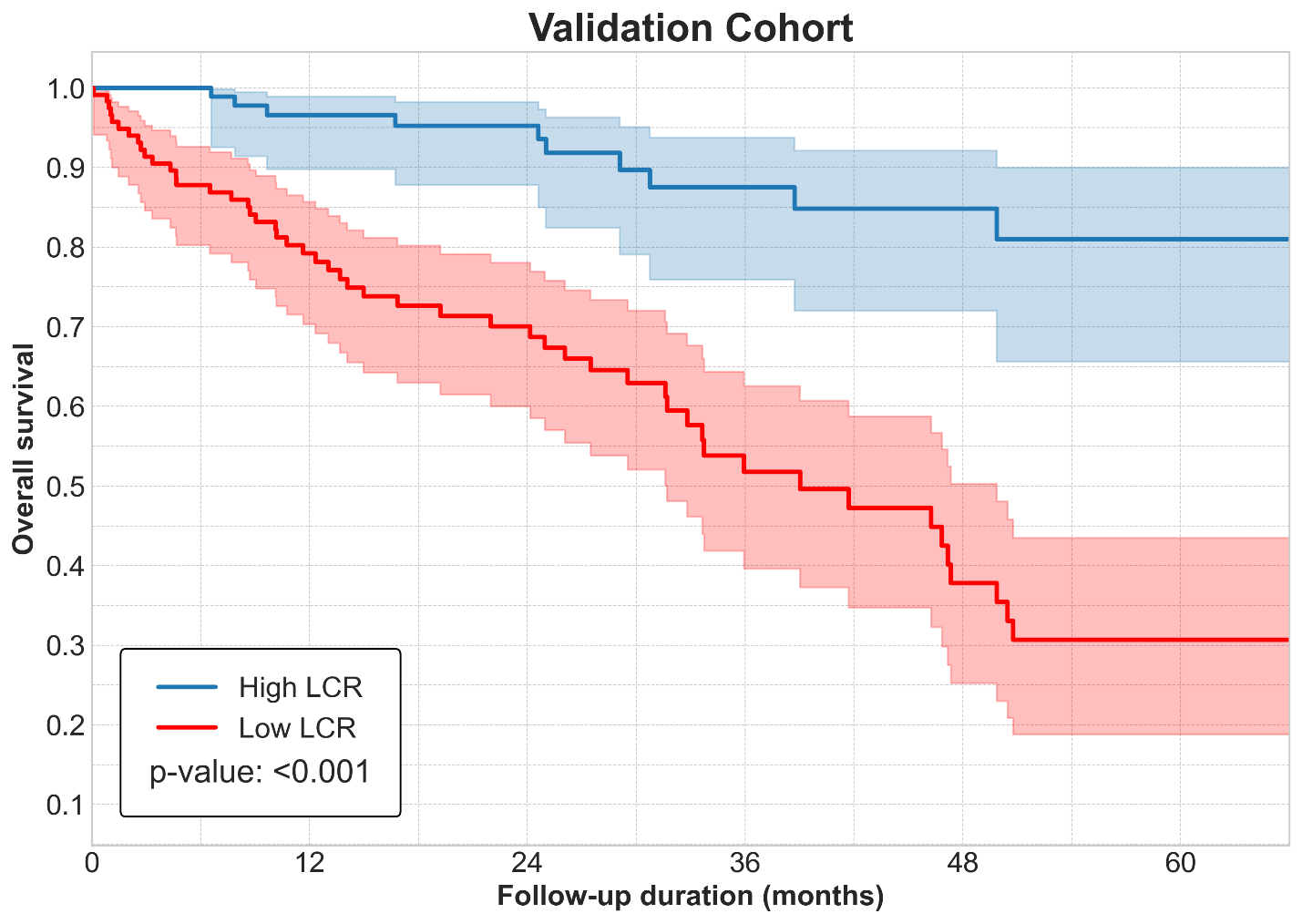


**Supplementary Table 1.** Calculation formulas for nine inflammatory biomarker combinations based on five key inflammatory markers—CRP, lymphocyte, neutrophil, platelet, and albumin.

| Inflammatory Biomarker | Calculation Formula |
| --- | --- |
| Lymphocyte-CRP ratio | Lymphocyte (count/µL) / CRP (mg/dL) |
| Neutrophil-Lymphocyte Ratio | Neutrophil (count/µL) / Lymphocyte (count/µL) |
| Platelet-Lymphocyte Ratio | Platelet (count/µL) / Lymphocyte (count/µL) |
| CRP-Albumin Ratio | CRP (mg/dl) / Albumin (g/dL) |
| Neutrophil x Platelet count | Neutrophil (count/µL) x Platelet (count/µL) |
| Neutrophil count x CRP | Neutrophil (count/µL) x CRP (mg/dL) |
| Platelet count x CRP | Platelet (count/µL) x CRP (mg/dL) |
| Platelet-Albumin Ratio | Platelet (count/µL) / Albumin (g/dL) |
| Lymphocyte count x Albumin | Lymphocyte (count/µL) x Albumin (g/dL) |

^Abbreviations:^ **^CRP:^** ^C-reactive protein.^

**Supplementary Table 2.** Univariable and multivariable logistic regression analyses for factors associated with postoperative complications in the derivation cohort.

| Variables | Univariate analysis | | Multivariate analysis | |
| --- | --- | --- | --- | --- |
|  | OR 95%CI | *P* value | OR 95%CI | *P* value |
| Age  Sex, male  Diagnosis  HCC  ICC  ASA class, >2  Cirrhosis  ALBI grade, 2/3  LCR, <6100  Tumor grade  Well/Moderately differentiated  Poorly/Undifferentiated  T status  1/2  3/4  N status  N0  N1/Nx  MIS, yes  Operation time (per 10 mins)  Blood loss (per 100 mL) | 0.99 (0.98-1.01)  0.68 (0.45-1.02)  Ref.  3.80 (2.56-5.64)  1.94 (1.29-2.91)  0.61 (0.41-0.93)  3.16 (1.81-5.51)  2.45 (1.67-3.60)  Ref.  2.69 (1.78-4.10)  Ref.  3.18 (2.00-5.05)  Ref.  1.32 (1.08-1.61)  0.72 (0.46-1.14)  1.04 (1.03-1.06)  1.10 (1.06-1.14) | 0.462  0.060  <0.001  0.001  0.021  <0.001  <0.001  0.001  <0.001  0.007  0.162  <0.001  <0.001 | Ref.  2.92 (1.74-4.91)  2.07 (1.29-3.33)  1.25 (0.74-2.11)  2.11 (0.93-4.59)  1.98 (1.27-3.10)  Ref.  1.31 (0.73-2.34)  Ref.  1.79 (1.06-2.43)  Ref.  1.29 (0.99-1.67)  1.03 (1.01-1.04)  1.05 (1.02-1.09) | <0.001  0.003  0.406  0.059  0.003  0.370  0.042  0.058  0.001  0.003 |

^Abbreviations:^ **^HCC^**^: Hepatocellular carcinoma,^ **^ICC^**^: Intrahepatic cholangiocarcinoma,^ **^ASA^**^: American Society of Anesthesiologists,^ **^ALBI^**^: Albumin-Bilirubin,^ **^LCR^**^: Lymphocyte-C-reactive protein ratio,^ **^MIS^**^: Minimally invasive surgery.^

**Supplementary Table 3.** Univariable and multivariable logistic regression analyses for factors associated with postoperative infectious complications in the derivation cohort.

| Variables | Univariate analysis | | Multivariate analysis | |
| --- | --- | --- | --- | --- |
|  | OR 95%CI | *P* value | OR 95%CI | *P* value |
| Age  Sex, male  Diagnosis  HCC  ICC  ASA class, >2  Cirrhosis  ALBI grade, 2/3  LCR, <6100  Tumor grade  Well/Moderately differentiated  Poorly/Undifferentiated  T status  1/2  3/4  N status  N0  N1/Nx  MIS, yes  Operation time (per 10 mins)  Blood loss (per 100 mL) | 1.01 (0.98-1.02)  0.58 (0.35-0.96)  Ref.  3.67 (2.21-6.09)  2.20 (1.33-3.64)  0.74 (0.43-1.27)  3.74 (1.58-6.65)  3.16 (1.86-5.37)  Ref.  2.97 (1.65-5.31)  Ref.  1.67 (0.95-2.95)  Ref.  1.15 (0.89-1.48)  0.66 (0.36-1.23)  1.03 (1.02-1.04)  1.07 (1.04-1.11) | 0.863  0.035  <0.001  0.002  0.268  0.003  <0.001  <0.001  0.076  0.297  0.189  <0.001  <0.001 | 0.66 (0.36-1.19)  Ref.  2.02 (1.12-3.64)  2.34 (1.33-4.10)  2.46 (0.83-6.24)  2.80 (1.57-5.01)  Ref.  1.66 (0.80-3.46)   - 1. (1.00-1.03)   1.05 (1.02-1.09) | 0.170  0.019  0.003  0.103  <0.001  0.172  0.041  0.001 |

^Abbreviations:^ **^HCC^**^: Hepatocellular carcinoma,^ **^ICC^**^: Intrahepatic cholangiocarcinoma,^ **^ASA^**^: American Society of Anesthesiologists,^ **^ALBI^**^: Albumin-Bilirubin,^ **^LCR^**^: Lymphocyte-C-reactive protein ratio,^ **^MIS^**^: Minimally invasive surgery.^

**Supplementary Table 4.** Univariable and multivariable Cox regression analyses for factors associated with recurrence-free survival in the derivation cohort.

| Variables | Univariate analysis | | Multivariate analysis | |
| --- | --- | --- | --- | --- |
|  | HR 95%CI | *P* value | HR 95%CI | *P* value |
| Age  Sex, male  Diagnosis  HCC  ICC  ASA class, >2  Cirrhosis  Tumor marker  Low  Medium  High  ALBI grade, 2/3  LCR, <6100  Tumor grade  Well/Moderately differentiated  Poorly/Undifferentiated  Perineural invasion  Location, Bilobar  T status  1/2  3/4  N status  N0  N1/Nx  Resection margin, R1 | 0.99 (0.98-1.01)  0.77 (0.58-1.01)  Ref.  1.83 (1.40-2.39)  1.34 (1.01-1.78)  0.83 (0.62-1.11)  Ref.  0.90 (0.65-1.24)  0.84 (0.61-1.16)  1.61 (1.12-2.30)  3.21 (2.09-4.87)  Ref.  1.96 (1.47-2.63)  2.03 (1.51-2.73)  1.37 (1.00-1.87)  Ref.  2.47 (1.84-3.32)  Ref.  1.67 (1.45-1.91)  2.04 (1.46-2.84) | 0.376  0.059  <0.001  0.044  0.201  0.512  0.292  0.009  <0.001  <0.001  0.001  0.050  <0.001  0.003  <0.001 | Ref.  1.64 (1.15-2.30)  1.14 (0.85-1.53)  1.40 (0.88-2.22)  2.43 (1.41-3.83)  Ref.  1.53 (1.07-2.18)  0.96 (0.67-1.37)  Ref.  1.61 (1.18-2.28)  Ref.  1.61 (1.37-1.90)  1.29 (0.89-1.86) | 0.005  0.398  0.151  0.002  0.021  0.815  <0.001  0.005  0.177 |

^Abbreviations:^ **^HCC^**^: Hepatocellular carcinoma,^ **^ICC^**^: Intrahepatic cholangiocarcinoma,^ **^ASA^**^: American Society of Anesthesiologists,^ **^ALBI^**^: Albumin-Bilirubin,^ **^LCR^**^: Lymphocyte-C-reactive protein ratio.^

**Supplementary Table 5.** Univariable and multivariable Cox regression analyses for factors associated with overall survival in the derivation cohort.

| Variables | Univariate analysis | | Multivariate analysis | |
| --- | --- | --- | --- | --- |
|  | HR 95%CI | *P* value | HR 95%CI | *P* value |
| Age  Sex, male  Diagnosis  HCC  ICC  ASA class, >2  Cirrhosis  Tumor marker  Low  Medium  High  ALBI grade, 2/3  LCR, <6100  Tumor grade  Well/Moderately differentiated  Poorly/Undifferentiated  Perineural invasion  Location, Bilobar  T status  1/2  3/4  N status  N0  N1/Nx  Resection margin, R1 | 0.99 (0.98-1.01)  0.83 (0.61-1.14)  Ref.  2.35 (1.75-3.16)  1.37 (1.01-1.86)  0.86 (0.63-1.19)  Ref.  1.23 (0.84-1.79)  1.31 (0.91-1.89)  1.12 (0.79-1.57)  3.63 (2.61-5.05)  Ref.  1.16 (0.86-1.56)  2.24 (1.63-3.09)  2.01 (1.47-2.76)  Ref.  2.07 (1.50-2.88)  Ref.  1.19 (1.02-1.40)  1.63 (1.11-2.41) | 0.243  0.252  <0.001  0.047  0.376  0.284  0.145  0.526  <0.001  0.340  0.001  <0.001  <0.001  0.026  0.014 | Ref.  1.88 (1.32-2.67)  1.32 (0.95-1.81)  2.95 (2.10-4.16)  1.46 (0.99-2.17)  1.64 (1.14-2.35)  Ref.  1.25 (1.05-1.48)  Ref.  1.12 (0.85-1.27)  0.87 (0.57-1.35) | <0.001  0.095  <0.001  0.058  0.007  0.010  0.125  0.545 |

^Abbreviations:^ **^HCC^**^: Hepatocellular carcinoma,^ **^ICC^**^: Intrahepatic cholangiocarcinoma,^ **^ASA^**^: American Society of Anesthesiologists,^ **^ALBI^**^: Albumin-Bilirubin,^ **^LCR^**^: Lymphocyte-C-reactive protein ratio.^

**Supplementary Table 6.** Univariable and multivariable Cox regression analyses for factors associated with recurrence-free survival among patients with hepatocellular carcinoma.

| Variables | Univariate analysis | | Multivariate analysis | |
| --- | --- | --- | --- | --- |
|  | HR 95%CI | *P* value | HR 95%CI | *P* value |
| Age  Sex, male  ASA class, >2  Cirrhosis  Tumor marker  Low  Medium  High  ALBI grade, 2/3  LCR, <6100  Tumor grade  Well/Moderately differentiated  Poorly/Undifferentiated  Perineural invasion  Location, Bilobar  T status  1/2  3/4  N status  N0  N1/Nx  Resection margin, R1 | 1.01 (0.98-1.02)  0.84 (0.53-1.34)  1.12 (0.74-1.69)  0.98 (0.67-1.43)  Ref.  1.17 (0.75-1.83)  1.56 (1.08-2.38)  1.83 (1.16-2.92)  2.99 (1.94-4.36)  Ref.  2.88 (1.92-4.31)  2.26 (1.31-3.88)  2.07 (1.18-3.64)  Ref.  3.11 (1.99-4.86)  Ref.  2.13 (1.72-2.65)  1.96 (1.07-3.58) | 0.786  0.471  0.594  0.920  0.286  0.042  0.010  <0.001  <0.001  0.003  0.011  <0.001  <0.001  0.029 | Ref.  0.99 (0.63-1.57)  1.26 (0.74-2.13)  1.26 (0.64-2.46)  2.23 (1.56-3.54)  Ref.  1.68 (0.87-3.24)  1.46 (0.82-2.58)  1.18 (0.63-2.21)  Ref.  1.79 (1.38-2.32)  Ref.  1.78 (1.09-2.92)  0.95 (0.49-1.83) | 0.982  0.390  0.508  0.001  0.121  0.200  0.596  <0.001  0.021  0.872 |

^Abbreviations:^ **^ASA^**^: American Society of Anesthesiologists,^ **^ALBI^**^: Albumin-Bilirubin,^ **^LCR^**^: Lymphocyte-C-reactive protein ratio.^

**Supplementary Table 7.** Univariable and multivariable Cox regression analyses for factors associated with overall survival among patients with hepatocellular carcinoma.

| Variables | Univariate analysis | | Multivariate analysis | |
| --- | --- | --- | --- | --- |
|  | HR 95%CI | *P* value | HR 95%CI | *P* value |
| Age  Sex, male  ASA class, >2  Cirrhosis  AFP  Low  Medium  High  ALBI grade, 2/3  LCR, <6100  Tumor grade  Well/Moderately differentiated  Poorly/Undifferentiated  Perineural invasion  Location, Bilobar  T status  1/2  3/4  N status  N0  N1/Nx  Resection margin, R1 | 0.99 (0.97-1.01)  1.44 (0.74-2.79)  1.01 (0.61-1.67)  1.19 (0.75-1.86)  Ref.  1.05 (0.60-1.83)  1.19 (0.68-2.10)  1.39 (0.87-2.21)  4.08 (2.15-6.02)  Ref.  0.99 (0.61-1.60)  1.94 (0.87-4.31)  3.39 (1.92-5.98)  Ref.  2.38 (1.35-4.22)  Ref.  1.29 (1.02-1.62)  1.61 (0.70-3.74) | 0.414  0.288  0.975  0.462  0.871  0.546  0.166  <0.001  0.970  0.105  <0.001  0.003  0.036  3.74 | 3.85 (2.05-5.40)  1.09 (0.57-2.10)  Ref.  2.55 (1.34-4.85)  Ref.  1.08 (0.85-1.37) | <0.001  0.790  0.004  0.515 |

^Abbreviations:^ **^ASA^**^: American Society of Anesthesiologists,^ **^ALBI^**^: Albumin-Bilirubin,^ **^LCR^**^: Lymphocyte-C-reactive protein ratio.^

**Supplementary Table 8.** Univariable and multivariable Cox regression analyses for factors associated with recurrence-free survival among patients with intrahepatic cholangiocarcinoma.

| Variables | Univariate analysis | | Multivariate analysis | |
| --- | --- | --- | --- | --- |
|  | HR 95%CI | *P* value | HR 95%CI | *P* value |
| Age  Sex, male  ASA class, >2  Cirrhosis  CA 19-9  Low  Medium  High  ALBI grade, 2/3  Neoadjuvant therapy  LCR, <6100  Tumor grade  Well/Moderately differentiated  Poorly/Undifferentiated  Perineural invasion  Location, Bilobar  T status  1/2  3/4  N status  N0  N1/Nx  Lymph node dissection  Biliary reconstruction  Vascular resection  Resection margin, R1  Adjuvant chemotherapy | 0.99 (0.97-1.01)  0.98 (0.68-1.42)  1.72 (1.16-2.55)  1.17 (0.66-2.05)  Ref.  0.99 (0.63-1.58)  1.20 (0.77-1.88)  3.12 (1.70-5.72)  1.14 (0.55-2.35)  3.89 (2.24-5.71)  Ref.  1.74 (1.14-2.65)  1.40 (0.97-2.04)  0.83 (0.56-1.22)  Ref.  1.66 (1.12-2.47)  Ref.  1.52 (1.22-1.90)  1.24 (1.05-1.44)  1.21 (1.02-1.42)  1.10 (0.92-1.34)  1.62 (1.07-2.44)  0.95 (0.62-1.44) | 0.317  0.929  0.007  0.591  0.981  0.416  <0.001  0.722  <0.001  0.010  0.076  0.350  0.011  0.001  0.003  0.022  0.289  0.022  0.801 | 1.34 (0.89-2.04)  2.78 (1.49-5.19)  2.72 (1.36-4.86)  Ref.  1.27 (0.77-2.11)  Ref.  1.34 (1.05-1.70)  Ref.   - 1. (0.64-1.60)   1.04 (0.83-1.38)  1.12 (0.87-1.52)  1.17 (0.76-1.79) | 0.162  0.001  0.006  0.347  0.018  0.543  0.315  0.285  0.473 |

^Abbreviations:^ **^ASA^**^: American Society of Anesthesiologists,^ **^ALBI^**^: Albumin-Bilirubin,^ **^LCR^**^: Lymphocyte-C-reactive protein ratio.^

**Supplementary Table 9.** Univariable and multivariable Cox regression analyses for factors associated with overall survival among patients with intrahepatic cholangiocarcinoma.

| Variables | Univariate analysis | | Multivariate analysis | |
| --- | --- | --- | --- | --- |
|  | HR 95%CI | *P* value | HR 95%CI | *P* value |
| Age  Sex, male  ASA class, >2  Cirrhosis  CA 19-9  Low  Medium  High  ALBI grade, 2/3  Neoadjuvant therapy  LCR, <6100  Tumor grade  Well/Moderately differentiated  Poorly/Undifferentiated  Perineural invasion  Location, Bilobar  T status  1/2  3/4  N status  N0  N1/Nx  Lymph node dissection  Biliary reconstruction  Vascular resection  Resection margin, R1  Adjuvant therapy | 0.99 (0.98-1.01)  1.00 (0.68-1.47)  1.69 (1.14-2.51)  1.39 (0.81-2.36)  Ref.  1.47 (0.88-2.45)  1.97 (1.21-3.20)  3.05 (1.70-5.48)  1.34 (0.65-2.77)  2.57 (1.67-3.97)  Ref.  1.62 (1.06-2.49)  1.61 (1.10-2.36)  1.09 (0.74-1.61)  Ref.  1.50 (1.20-2.05)  Ref.  1.38 (1.10-1.74)  0.96 (0.81-1.15)  0.96 (0.79-1.18)  0.92 (0.75-1.14)  1.17 (0.75-1.83)  0.65 (0.40-1.07) | 0.556  0.989  0.009  0.227  0.145  0.006  <0.001  0.429  <0.001  0.026  0.014  0.661  0.002  0.005  0.688  0.728  0.472  0.495  0.091 | 1.38 (0.91-2.10)  Ref.  1.27 (0.75-2.14)  1.44 (1.06-2.18)  2.99 (1.62-5.52)  2.14 (1.37-3.35)  Ref.  1.24 (0.76-2.00)  1.30 (0.85-1.99)  Ref.  1.24 (1.10-1.45)  Ref.  0.95 (0.59-1.54) | 0.135  0.377  0.022  <0.001  0.001  0.390  0.229  0.027  0.838 |

^Abbreviations:^ **^ASA^**^: American Society of Anesthesiologists,^ **^ALBI^**^: Albumin-Bilirubin,^ **^LCR^**^: Lymphocyte-C-reactive protein ratio.^

**Supplementary Table 10.** Comparison of ROC Curves for recurrence-free and overall survival of different inflammatory biomarkers relative to Lymphocyte-CRP ratio.

| Inflammatory Marker for Comparison | ROC Curve Analysis for Recurrence-free survival | | | ROC Curve Analysis for Overall survival | | |
| --- | --- | --- | --- | --- | --- | --- |
|  | SE of difference | *Z* statistic | *P* value | SE of difference | *Z* statistic | *P* value |
| Neutrophil-Lymphocyte Ratio | 0.038 | 1.521 | 0.128 | 0.043 | 2.359 | 0.018 |
| Platelet-Lymphocyte Ratio | 0.038 | 2.188 | 0.029 | 0.043 | 2.567 | 0.010 |
| CRP-Albumin Ratio | 0.038 | 1.151 | 0.250 | 0.042 | 0.298 | 0.765 |
| Neutrophil x Platelet count | 0.039 | 3.868 | <0.001 | 0.041 | 1.937 | 0.043 |
| Neutrophil count x CRP | 0.038 | 1.870 | 0.061 | 0.043 | 0.789 | 0.430 |
| Platelet count x CRP | 0.038 | 1.666 | 0.096 | 0.043 | 0.894 | 0.371 |
| Platelet-Albumin Ratio | 0.038 | 6.701 | <0.001 | 0.041 | 7.553 | <0.001 |
| Lymphocyte count x Albumin | 0.039 | 2.276 | 0.023 | 0.040 | 3.579 | 0.003 |

**Supplementary Table 11.** Comparison of Lymphocyte-C-reactive protein ratio across clinicodemographic variables in the validation cohort.

| Variable | Number (%) | Median LCR | IQR | *P* value |
| --- | --- | --- | --- | --- |
| Age  <65 years  ≥65 years | 113 (52.3%)  103 (47.7%) | 5213.0  4564.8 | 36741.1  25722.2 | 0.623 |
| Sex  Male  Female | 152 (70.4%)  64 (29.6%) | 5062.9  4069.4 | 36196.8  31759.5 | 0.203 |
| ASA class  ≤2  >2 | 154 (71.3%)  62 (28.7%) | 5642.7  3979.0 | 88784.0  10848.0 | 0.066 |
| Tumor marker  Low  Medium  High | 72 (33.3%)  76 (35.2%)  68 (31.5%) | 5536.2  5062.9  4292.7 | 30166.8  27658.2  43888.3 | 0.744 |
| TBS  Low  Medium  High | 80 (37.0%)  69 (31.9%)  67 (31.0%) | 10372.6  5180.2  2346.9 | 93062.2  92164.6  10596.2 | 0.001 |
| ALBI grade  1  2/3 | 179 (82.9%)  37 (17.1%) | 5002.8  1923.1 | 33101.7  33727.4 | 0.143 |
| Major vascular invasion  Absent  Present | 190 (88.0%)  26 (12.0%) | 5547.7  3035.7 | 44903.0  4629.2 | 0.064 |
| Tumor grade  Well/moderately differentiated  Poorly/undifferentiated | 133 (61.6%)  83 (38.4%) | 5213.0  4600.0 | 24057.3  10182.1 | 0.737 |
| Perineural invasion  Absent  Present | 176 (81.5%)  40 (18.5%) | 5967.5  4292.7 | 90421.5  6644.2 | 0.010 |
| Resection margin  R0  R1 | 192 (88.9%)  22 (10.1%) | 5882.4  1438.5 | 77872.8  4172.6 | 0.011 |
| T status  I/II  III/IV | 178 (82.4%)  38 (17.6%) | 5196.6  2001.9 | 77646.4  10709.9 | 0.014 |
| N status  N0  N1/Nx | 131 (60.6%)  85 (39.4%) | 7707.0  4210.0 | 94466.2  8557.5 | 0.040 |

^Abbreviations:^ **^ASA^**^: American Society of Anesthesiologists,^ **^TBS^**^: Tumor burden score,^ **^ALBI^**^: Albumin-Bilirubin,^ **^LCR^**^: Lymphocyte-C-reactive protein ratio,^ **^IQR^**^: Interquartile range.^

**Supplementary Table 12.** Univariable and multivariable logistic regression analyses for factors associated with postoperative complications in the validation cohort

| Variables | Univariate analysis | | Multivariate analysis | |
| --- | --- | --- | --- | --- |
|  | OR 95%CI | *P* value | OR 95%CI | *P* value |
| Age  Sex, male  Diagnosis  HCC  ICC  ASA class, >2  Cirrhosis  ALBI grade, 2/3  LCR, <6100  Tumor grade  Well/Moderately differentiated  Poorly/Undifferentiated  T status  1/2  3/4  N status  N0  N1/Nx  MIS, yes  Operation time (per 10 mins)  Blood loss (per 100 mL) | 1.02 (1.00-1.05)  0.46 (0.25-0.85)  Ref.  3.28 (1.81-6.00)  1.58 (0.85-2.93)  0.42 (0.21-0.80)  4.59 (1.56-6.41)  3.03 (1.63-5.64)  Ref.  3.00 (1.56-5.75)  Ref.  2.304 (1.13-4.71)  Ref.  1.63 (1.19-2.21)  0.33 (0.15-0.72)  1.08 (1.05-1.11)  1.10 (1.05-1.15) | 0.108  0.013  <0.001  0.148  0.009  0.006  <0.001  0.001  0.022  0.002  0.005  <0.001  <0.001 | 0.70 (0.31-1.58)  Ref.  2.05 (1.14-4.79)  0.52 (0.21-1.27)  3.09 (0.65-5.95)  2.08 (1.07-4.35)  Ref.  1.73 (0.72-4.17)  Ref.  1.69 (0.69-4.12)  Ref.  1.63 (1.03-2.56)  0.37 (0.14-0.95)  1.05 (0.99-1.10)  1.05 (1.02-1.09) | 0.395  0.014  0.153  0.154  0.039  0.221  0.249  0.035  0.038  0.091  0.001 |

^Abbreviations:^ **^HCC^**^: Hepatocellular carcinoma,^ **^ICC^**^: Intrahepatic cholangiocarcinoma,^ **^ASA^**^: American Society of Anesthesiologists,^ **^ALBI^**^: Albumin-Bilirubin,^ **^LCR^**^: Lymphocyte-C-reactive protein ratio,^ **^MIS^**^: Minimally invasive surgery.^

**Supplementary Table 13.** Univariable and multivariable logistic regression analyses for factors associated with postoperative infectious complications in the validation cohort.

| Variables | Univariate analysis | | Multivariate analysis | |
| --- | --- | --- | --- | --- |
|  | OR 95%CI | *P* value | OR 95%CI | *P* value |
| Age  Sex, male  Diagnosis  HCC  ICC  ASA class, >2  Cirrhosis  ALBI grade, 2/3  LCR, <6100  Tumor grade  Well/Moderately differentiated  Poorly/Undifferentiated  T status  1/2  3/4  N status  N0  N1/Nx  MIS, yes  Operation time (per 10 mins)  Blood loss (per 100 mL) | 1.01 (0.98-1.04)  0.31 (0.15-0.64)  Ref.  3.76 (1.82-7.77)  1.51 (0.72-3.13)  0.59 (0.27-1.30)  2.01 (0.67-6.04)  6.70 (2.88-9.60)  Ref.  3.24 (1.69-5.26)  Ref.   - 1. (0.42-2.55)   Ref.  1.16 (0.80-1.69)  0.27 (0.09-0.81)  1.07 (1.03-1.10)  1.10 (1.05-1.15) | 0.539  0.001  <0.001  0.275  0.191  0.216  <0.001  0.002  0.949  0.420  0.019  <0.001  <0.001 | 0.46 (0.17-1.23)  Ref.  1.59 (0.60-4.21)  5.81 (2.28-7.36)  Ref.  2.98 (1.58-5.05)  0.35 (0.09-1.29)  1.04 (1.01-1.08)  1.08 (1.02-1.13) | 0.123  0.355  0.001  0.002  0.115  0.019  0.006 |

^Abbreviations:^ **^HCC^**^: Hepatocellular carcinoma,^ **^ICC^**^: Intrahepatic cholangiocarcinoma,^ **^ASA^**^: American Society of Anesthesiologists,^ **^ALBI^**^: Albumin-Bilirubin,^ **^LCR^**^: Lymphocyte-C-reactive protein ratio,^ **^MIS^**^: Minimally invasive surgery.^

**Supplementary Table 14.** Univariable and multivariable Cox regression analyses for factors associated with recurrence-free survival in the validation cohort.

| Variables | Univariate analysis | | Multivariate analysis | |
| --- | --- | --- | --- | --- |
|  | HR 95%CI | *P* value | HR 95%CI | *P* value |
| Age  Sex, male  Diagnosis  HCC  ICC  ASA class, >2  Cirrhosis  Tumor marker  Low  Medium  High  ALBI grade, 2/3  LCR, <6100  Tumor grade  Well/Moderately differentiated  Poorly/Undifferentiated  Perineural invasion  Location, Bilobar  T status  1/2  3/4  N status  N0  N1/Nx  Resection margin, R1 | 0.99 (0.97-1.01)  0.88 (0.55-1.41)  Ref.  1.96 (1.25-3.09)  1.94 (1.22-3.07)  0.79 (0.48-1.31)  Ref.  1.20 (0.70-2.10)  1.02 (0.58-1.77)  0.64 (0.33-1.25)  3.24 (1.20-6.86)  Ref.  1.76 (1.06-2.92)  1.62 (0.95-2.75)  1.95 (1.19-3.17)  Ref.  3.32 (2.06-5.34)  Ref.  1.71 (1.35-2.17)  2.58 (1.46-4.57) | 0.412  0.590  0.004  0.005  0.364  0.514  0.955  0.194  <0.001  0.029  0.074  0.007  <0.001  <0.001  <0.001 | Ref.  1.77 (1.01-3.13)  2.01 (1.24-3.24)  2.89 (1.39-5.57)  Ref.  1.11 (0.63-1.95)  Ref.  1.98 (1.19-3.29)  Ref.  1.83 (1.37-2.44)  1.37 (0.74-2.56) | 0.049  0.005  0.002  0.529  <0.001  0.009  0.317 |

^Abbreviations:^ **^HCC^**^: Hepatocellular carcinoma,^ **^ICC^**^: Intrahepatic cholangiocarcinoma,^ **^ASA^**^: American Society of Anesthesiologists,^ **^ALBI^**^: Albumin-Bilirubin,^ **^LCR^**^: Lymphocyte-C-reactive protein ratio.^

**Supplementary Table 15.** Univariable and multivariable Cox regression analyses for factors associated with overall survival in the validation cohort.

| Variables | Univariate analysis | | Multivariate analysis | |
| --- | --- | --- | --- | --- |
|  | HR 95%CI | *P* value | HR 95%CI | *P* value |
| Age  Sex, male  Diagnosis  HCC  ICC  ASA class, >2  Cirrhosis  Tumor marker  Low  Medium  High  ALBI grade, 2/3  LCR, <6100  Tumor grade  Well/Moderately differentiated  Poorly/Undifferentiated  Perineural invasion  Location, Bilobar  T status  1/2  3/4  N status  N0  N1/Nx  Resection margin, R1 | - 1. (0.98-1.02)   0.84 (0.50-1.41)  Ref.  1.82 (1.10-3.02)  1.59 (0.95-2.66)  1.01 (0.59-1.73)  Ref.  1.21 (0.63-2.34)  1.62 (0.87-3.02)  1.36 (0.75-2.48)  3.50 (1.29-5.85)  Ref.  1.17 (0.70-1.94)  2.02 (1.14-3.57)  2.24 (1.33-3.78)  Ref.  2.64 (1.56-4.47)  Ref.  1.31 (1.00-1.72)  1.84 (0.96-3.54) | 0.879  0.506  0.020  0.079  0.963  0.574  0.131  0.313  <0.001  0.550  0.016  0.002  <0.001  0.049  0.066 | Ref.  1.50 (0.80-2.81)  2.96 (1.15-4.42)  1.28 (0.65-2.54)  1.86 (1.02-3.37)  Ref.  1.84 (1.20-3.32)  Ref.  1.38 (1.02-1.86) | 0.204  0.001  0.472  0.040  0.037  0.043 |

^Abbreviations:^ **^HCC^**^: Hepatocellular carcinoma,^ **^ICC^**^: Intrahepatic cholangiocarcinoma,^ **^ASA^**^: American Society of Anesthesiologists,^ **^ALBI^**^: Albumin-Bilirubin,^ **^LCR^**^: Lymphocyte-C-reactive protein ratio.^
